# siRNA-Mediated B7H7 Knockdown in Gastric Cancer Lysate-Loaded Dendritic Cells Amplifies Expansion and Cytokine Secretion of Autologous T Cells

**DOI:** 10.3390/biomedicines11123212

**Published:** 2023-12-04

**Authors:** Javad Masoumi, Farid Ghorbaninezhad, Hossein Saeedi, Sahar Safaei, Vahid Khaze Shahgoli, Amir Ghaffari Jolfayi, Bahar Naseri, Amir Baghbanzadeh, Elham Baghbani, Ahad Mokhtarzadeh, Mohammad Bakhshivand, Mohammad Reza Javan, Nicola Silvestris, Behzad Baradaran

**Affiliations:** 1 Immunology Research Center, Tabriz University of Medical Sciences, Tabriz 51548-53431, Iran; masoumi.javad.2024@gmail.com (J.M.); ghorbaninezhadfarid@gmail.com (F.G.); khazev@tbzmed.ac.ir (V.K.S.); amirghaffari1996@gmail.com (A.G.J.); mrsb7696@gmail.com (B.N.); amirbaghbanzadeh@gmail.com (A.B.); mokhtarzadehah@tbzmed.ac.ir (A.M.);; 2 Student Research Committee, Tabriz University of Medical Sciences, Tabriz 51548-53431, Iran; 3 Pharmaceutical Analysis Research Center, Tabriz University of Medical Sciences, Tabriz 51548-53431, Iran; 4 Department of Immunology, Faculty of Medicine, Zabol University of Medical Sciences, Zabol 98616-15881, Iran; m.r.javan@zbmu.ac.ir; 5 Medical Oncology Unit, Department of Human Pathology “G. Barresi”, University of Messina, 98122 Messina, Italy; 6 Department of Immunology, Faculty of Medicine, Tabriz University of Medical Sciences, Tabriz 51548-53431, Iran

**Keywords:** gastric cancer, dendritic cell, T cell, B7H7, DC-based cell therapy

## Abstract

Background: Gastric cancer, ranked as the fifth most common cancer worldwide, presents multiple treatment challenges. These obstacles often arise due to cancer stem cells, which are associated with recurrence, metastasis, and drug resistance. While dendritic cell (DC)-based immunotherapy has shown promise as a therapeutic strategy, its efficacy can be limited by the tumor microenvironment and certain inhibitory immune checkpoint molecules, such as B7H7. SiRNA-medicated knockdown of B7H7 in tumor cell lysate-pulsed DCs can increase cytokine secretion and autologous T lymphocyte expansion. This study aimed to evaluate the impact of B7H7 suppression in gastric cancer cell lysate-pulsed DCs on the stimulatory potential of autologous CD3^+^ T lymphocytes. Methods: Peripheral blood mononuclear cells (PBMCs) were isolated and monocytes were obtained; then, they were differentiated to immature DCs (iDCs) by GM-CSF and IL-4. Tumor cell lysates from human gastric cancer cell lines were harvested, and iDCs were transformed into mature DCs (mDCs) by stimulating iDCs with tumor cell lysate and lipopolysaccharide. B7H7-siRNA was delivered into mDCs using electroporation, and gene silencing efficiency was assessed. The phenotypic characteristics of iDCs, mDCs, and B7H7-silenced mDCs were evaluated using specific surface markers, an inverted light microscope, and flow cytometry. CD3^+^ T cells were isolated via magnetically activated cell sorting. They were labeled with CFSE dye and co-cultured with mDCs and B7H7-silenced mDCs to evaluate their ability to induce T-cell proliferation. T-cell proliferation was assessed using flow cytometry. The concentration of TGF-β, IL-4, and IFN-γ secreted from CD3^+^ T cells in the co-cultured supernatant was evaluated to investigate the cytokine secretory activity of the cells. Results: Transfection of B7H7 siRNA into mDCs was performed in optimal conditions, and the siRNA transfection effectively reduced B7H7 mRNA expression in a dose-dependent manner. SiRNA-mediated B7H7 knockdown in mDCs enhanced maturation and activation of the DCs, as demonstrated by an increased surface expression of CD11c, CD86, and CD40. Co-culture experiments revealed that B7H7-silenced mDCs had more capacity to induce T cell proliferation compared to non-transfected mDCs. The cytokine production patterns of T cells were also altered. Upon examining the levels of TGF-β, IL-4, and IFN-γ released by CD3^+^ T cells in the co-culture supernatant, we found that silencing B7H7 in mDCs resulted in a rise in IL-4 secretion and a reduction in TGF-β levels compared to mDCs that were not transfected. Conclusions: The study found that suppressing B7H7 expression in DCs significantly enhances their maturation and stimulatory activity when exposed to gastric cancer cell lysate. These B7H7-silenced DCs can substantially increase cytokine production and promote co-cultured T-cell expansion. Consequently, inhibiting B7H7 in DCs may offer a practical strategy to enhance the ability of DCs to initiate T lymphocyte responses and improve the effectiveness of DC-based cell therapy for cancer patients.

## 1. Introduction

Gastric cancer (GC) is the fifth most prevalent cancer after lung, breast, colorectal, and prostate cancer. It accounts for the third most deaths among cancer patients worldwide based on GLOBOCAN 2020 statistics [1]. It is estimated that diagnosis and treatment of patients with GC in the early stage of malignancy development can reach a survival rate of up to 90% in the consequent five years [2,3]. Surgery, chemotherapy, and radiotherapy are the typical GC treatment options. Although combining these strategies has been associated with clinical benefits for GC patients, recurrence and metastasis are still two main obstacles in treating GC [4]. In line with this, about 50% of individuals are diagnosed with advanced GC, and these patients are not suitable candidates for surgery [2].

Furthermore, 40–60% of patients who receive radical resection exhibit recurrence [3]. Consequently, because of the side effects and limitations of conventional therapies, there has been a shift in focus towards immunotherapy methods. This includes dendritic cell (DC) based immunotherapy, which is now considered a viable therapeutic strategy for GC treatment [5]. 

DCs are the most effective antigen-presenting cells (APCs) that can stimulate T lymphocyte responses to specific antigens and contribute to the development of tumor-specific immunity [6]. Regarding tumor-specific responses, Th1 and Th2 cells and their related cytokines, including IFN-γ, TNF-α, and IL-4, are mostly considered anti-tumor elements. However, regulatory T cells that produce cytokines, such as TGF-β and IL-10, are recognized as components that promote tumor growth [7]. Regulatory T cells promote tumor growth mainly by inhibiting T cell-mediated elimination of tumor cells through secretion of IL-10 and/or TGF-β [8]. Furthermore, IL-10 produced by regulatory T cells activates the STAT3/BCL2 pathway in GC cells and induces their resistance to chemotherapy [9].

The tumor-associated antigens (TAAs) would be advantageous as immunotherapy targets since the immune responses they trigger primarily affect cancer cells carrying the antigenic epitopes, reducing the chance of unintended damage to healthy tissues [10]. An effective anti-cancer immune response requires the activation of antigen-specific T cells by DCs and the elimination of tumor cells via effector T cells [11]. In both experimental tumor models and clinical trials, DC-based cell therapies, e.g., tumor lysate-sensitized DCs [12], tumor antigen-derived peptide-pulsed DCs [6], neoantigen-loaded DC vaccines [13], as well as cancer stem cell mRNA-transfected DCs [14], have demonstrated promising findings in inducing anti-tumor T cell responses. Despite these advances, the use of DC vaccines in the treatment of GC has not shown many promising outcomes. For example, only one of the three participating patients in phase I/II clinical trial using a Wilms tumor 1 (WT1)-targeted DC vaccination responded effectively to the treatment [15]. In this regard, immunosuppressive factors of the tumor microenvironment (TME), particularly inhibitory immune checkpoints (ICPs), remain an obstacle to DC-based cell therapy strategies and their efficacy [16]. Tumor cells can escape the immune responses by overexpressing inhibitory ICPs on DCs located within the TME [16]. Increased expression of various inhibitory ICPs on DCs is associated with their dysfunction. For instance, the maturation of DCs and their antigen-presentation capability is diminished by elevated levels of cytotoxic T-lymphocyte–associated antigen 4 (CTLA-4) [17] and programmed cell death ligand 1 (PDL-1) [18]. Additionally, it has been demonstrated that the interactions between additional inhibitory ICPs, such as T-cell immunoglobulin and mucin domain-containing protein 3 (TIM-3) [19], lymphocyte activation gene-3 (LAG-3) [20], v-domain Ig suppressor of T-cell activation (VISTA) [21], and B- and T-lymphocyte attenuator (BTLA) [22], reduce the efficacy of DC function. Hence, the overexpression of inhibitory ICPs on DCs diminishes the effectiveness of DC-based immunotherapies [16]. 

HERV–H LTR-associating 2 (HHLA2, also called B7H7) and its receptor CD28H are two recently identified B7 family ligands [23,24]. The B7H7 molecule is the only member of the B7 family found in humans but not mice and shares a 23–33% amino acid sequence similarity with other B7 molecules [23,25]. An increasing number of investigations have shown that the expression of B7H7 is widely increased in GC [26,27,28]. For individuals with gastric cancer, B7H7 overexpression is associated with an advanced clinical stage, deep tumor invasion, metastasis, and limited overall survival, and it indicates a poor prognosis [27]. Regarding the expression of this molecule by immune cells, there is no detectable expression of B7H7 by inactive B and T cells or immature DCs (iDCs) in the human immune system, but it is highly expressed in macrophages and monocytes [29]. Following stimulation with inflammatory signals, i.e., poly I:C, interferon (IFN-γ), and lipopolysaccharide (LPS), B7H7 expression is elevated in B lymphocytes and enhanced in monocytes and mature DCs [23]. B7H7 exhibits both co-inhibitory and co-stimulatory properties on T lymphocytes [29]. In a study by Luu et al., it has been shown that B7H7 has a predominant expression by exhausted cytotoxic T lymphocytes (CTLs) and T helper 1 cells, which have impaired production of Tumor necrosis factor (TNF)-α and IFN-γ production [30]. In another study on NK-92MI and Jurkat T cells, it was revealed that an alternative receptor for B7H7 on T cells and NK cells is KIR3DL3 (Killer Cell Immunoglobulin Like Receptor), a protein containing three Ig domains with a long cytoplasmic tail. The binding of B7H7 to this receptor leads to a significant reduction in T-lymphocytes activation and NK cell cytotoxicity [31]. It has also been demonstrated that B7H7 suppresses the activation and proliferation of CD8^+^ and CD4^+^ T-cells through the modulation of T cell receptor (TCR) and CD28 signaling [32]. However, no study has been held on the function of B7H7 in DC-mediated anti-cancer immunity. 

**Figure 1:**
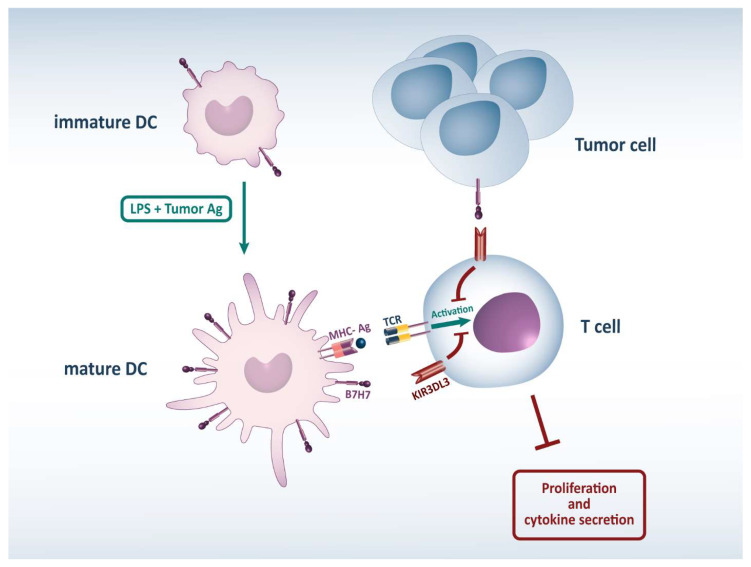
B7H7 inhibits T cells’ anti-tumor responses. B7H7 expressed by mature DCs and tumor cells by binding to KIR3DL3 on T cells and transmitting inhibitory signals can hamper their anti-tumor responses. Abbreviations: DC: Dendritic Cell, LPS: Lipopolysaccharides MHC: Major Histocompatibility Complex, Ag: Antigen, TCR: T Cell Receptor, KIR3DL3: Killer Cell Immunoglobulin Like Receptor.

Considering the inhibitory roles of the B7H7 molecule in reducing the function of T cells, its constitutive expression by monocytes, and the enhancement of its expression by monocytes and mature DCs with poly I:C, LPS, and IFN-γ, we assumed that B7H7 expression by monocytes-derived DCs is associated with the inhibition of T cell immunity initiated after DCs presented tumor-associated antigens to T cells (Figure 1).

In this study, we aimed to investigate the impact of suppressing B7H7 expression in dendritic cells (DCs) pulsed with GC cell lysate. By utilizing small interfering RNA (siRNA) to inhibit B7H7 expression in these DCs, we examined their phenotypic properties and stimulatory potential, focusing on T-cell responses in co-cultures of DCs with T lymphocytes. Our objective was to determine whether the suppression of B7H7 in GC cell lysate-pulsed DCs could enhance their stimulatory capacity and boost the cytokine secretion and expansion of autologous CD3^+^T lymphocytes. Based on our findings, we posit that B7H7-inhibited GC cell lysate-pulsed DCs offer significant promise in enhancing the efficacy of DC-based cell therapies.

## 2. Materials and Methods

### 2.1. Materials

In the present study, complete medium (CM) includes RPMI 1640 (Gibco, New York, NY, USA), which consists of 10% Fetal Bovine Serum (FBS) (Gibco, New York, NY, USA), penicillin 100 IU/mL (Gibco, New York, NY, USA), streptomycin 100 mg/mL, and 2 mmol/L of L-glutamine (Gibco, New York, NY, USA). Recombinant human granulocyte-macrophage colony-stimulating factor (rh GM-CSF) and recombinant human interleukin-4 (rh IL-4) were obtained from Sigma Chemical Co. (Munich, Germany) and eBioscience (San Diego, CA, USA), respectively. 2-mercaptoethanol (2ME) was purchased from Gibco (New York, NY, USA). The human pan T cell isolation kit was provided from MiltenyiBiotec, Bergisch, Germany. The Carboxyfluorescein succinimidyl ester (CFSE) kit for labeling the cells was purchased from BioLegend (San Diego, CA, USA). Bradford protein assay kit was purchased from Bio-Rad, (Hercules, CA, USA). Ficoll was purchased from Sigma Chemical Co. (Munich, Germany). Antibodies utilized to phenotype the cells included anti-HLA-DR-APC and anti-CD86-PerCP-cy5.5 from BioLegend (San Diego, CA, USA) and anti-CD40-CF-blue and anti-CD11c-FITC from Immunostep (Salamanca, Spain).

### 2.2. Tumor Cell Culture and Preparation of Tumor Lysate

The National Cell Bank of Iran (Pasteur Institute, Tehran, Iran) provided human GC cell lines, including AGS, KATO-III, and MKN-45 cells. Provided cells were cultured in a complete medium and incubated at 37 °C with 5% CO_2_ and humidification. After cultivated cells had reached a 70–80% confluency, they were separated using trypsin, washed in serum-free medium two times, and resuspended in sterile Phosphate Buffered Saline solution (PBS) with a concentration of 1 × 10^7^ cells/mL. Tumor cell lysates were produced from cell suspensions using six rapid freeze–thaw cycles in liquid nitrogen and then a water bath at a temperature of 37 °C. The generated lysate was next processed with a 15 s sonication procedure to increase the tumor antigens release from lysed cancerous cells. Then, the tumor cell lysates underwent centrifugation at 1500 rpm at 4 °C for 15 min to eliminate cellular debris. A 0.2 µm filter was used to filter the obtained supernatant. The Bradford assay measured the amount of protein in the lysates.

### 2.3. Isolation of Peripheral Blood Mononuclear Cells (PBMCs) and DCs Generation

Heparin-containing sterile falcons were used to collect fresh peripheral blood from healthy donors. PBMCs were then separated from the blood samples using Ficoll gradients fractionation. Monocytes were isolated from PBMCs using the plastic adherence approach. To achieve this, PBMCs were cultivated in 6-well plates at a concentration of 5 × 10^6^ cells/mL of serum-free RPMI-1640 media. After 2 h of incubation at 37 °C, cells that were not adherent were removed, and adherent cells were then cultivated in CM where 50 µM of 2-Mercaptoethanol, 20 ng/mL of rhIL-4, and 40 ng/mL of rhGM-CSF had been added. On days 2 and 4, fresh CM supplemented with rhGM-CSF and rhIL-4 was added to the remaining half of the media to feed the cultures. On day 6, iDCs were collected, and 80 µg/mL of mixed lysate from human GC cell lines was added to the growth media. Following 5 h of incubation at 37 °C, 100 ng/mL of LPS was added to the culture media. Following 24 h of incubation at 37 °C, mature DCs (mDCs) loaded with tumor cell lysate were developed. 

### 2.4. Characterization of DCs’ Morphology and Phenotype

Using an inverted light microscope (Optika, XDS-3, Ponteranica, Italy), the monocytes and DCs’ morphology were studied, and pictures were obtained. Surface markers, including CD11c (anti-CD11c-FITC), HLA-DR (anti-HLA-DR-APC), CD40 (anti-CD40-CF-blue), and CD86 (anti-CD86-PerCP-cy5.5), were used for labeling the iDCs, mDCs, and B7H7-silenced mDCs to investigate their phenotypic characteristics. The cells were evaluated via a MACSQuant cytometer (Miltenyi Biotec, Auburn, CA, USA), and the findings were then analyzed utilizing FlowJo v10.5.3 software.

### 2.5. siRNA Delivery in DCs by Electroporation and Gene Silencing

Transfection reagent, scramble siRNA, and B7H7-siRNA were purchased from Santa Cruz Biotechnology (Santa Cruz, Canada). Table 1 displays the purchased B7H7-siRNA sequence. mDCs were collected and transfected with several pulse voltages of 160, 180, and 200 Volts, based on previously published articles similar to our work, utilizing Gene Pulser Xcell (Bio-Rad, USA) to find the optimum pulse voltage for B7H7-siRNA transfection. The transfection effectiveness of siRNA at various pulse voltages was assessed with FITC-labeled control siRNA provided by Santa Cruz Biotechnology (Santa Cruz, Canada). After determining 160 Volts as the optimum pulse voltage for transfection, based on the company’s recommendation, 40 and 80 pmol concentrations of B7H7-siRNA, as well as scramble siRNA, were used to transfect mDCs. Subsequently, mDCs were placed into a 6-well CM-containing plate following electroporation. The relative expression of the B7H7 molecule was assessed after 72 h of incubation utilizing quantitative real-time PCR (qRT-PCR). Based on the obtained data, the optimum siRNA dosage and pulse voltage were established for subsequent experiments.

### 2.6. Isolation of Autologous CD3^+^ T Lymphocytes

The autologous CD3^+^ T cells were isolated from PBMCs of the same donors employed for DC development using magnetically activated cell sorting (MACS) via a human pan T Cell Isolation Kit following the manufacturer’s guidelines. In brief, the cell suspension was centrifuged at 300× *g* for 10 min after the isolation of PBMCs. The supernatant part was removed, and pan T cell biotin Ab cocktail (10 μL) and 40 μL of MACS buffer were added for every 1 × 10^7^ cells. After incubating at 2–8 °C for 5 min, MACS buffer (30 μL) and pan T Cell Microbead cocktail (20 μL) were added per 1 × 10^7^ total cells. Cells were washed with MACS buffer and, after 10 min of incubation at 2–8 °C, resuspended in 500 μL of MACS buffer. The cell suspension was loaded to the MACS column and placed in the magnetic field of the MACS separator. Cells moving over the column were considered unlabeled and negatively selected CD3^+^ T cells.

### 2.7. CD3^+^ T Lymphocytes’ CFSE Labeling and Proliferation Assay

Isolated CD3^+^ T cells were labeled with CFSE following the manufacturer’s instructions. Briefly, isolated T cells were resuspended in PBS and incubated for 5 min, at 25 °C temperature, in the dark, with 5 µM CFSE. By adding RPMI-1640 medium with 20% FBS, the reaction was interrupted. Then, the harvested cells were transferred into a pre-warmed culture medium after the final washing process. A co-culture of DCs and T cells was used to evaluate the mDCs and B7H7-silenced mDCs for their capacity to promote autologous T cell proliferation. In a V-bottom 96-well plate, mDCs and B7H7-silenced-mDCs were co-cultured in the ratios of 1:5 and 1:10 with autologous CD3^+^ T lymphocytes labeled with CFSE as responder cells. The positive control included T cells triggered by phytohemagglutinin 5% (Sigma Chemical Co., Munich, Germany), whereas an unstimulated group included co-cultured T cells with iDCs. The CFSE-labeled T cells’ proliferation was investigated using flow cytometry after four days of incubation under dark conditions. Unstained CD3^+^ T lymphocytes were utilized as the unstained group.

### 2.8. RNA Extraction and qRT-PCR

The TRIzol reagent (Roche Diagnostics, Mannheim, Germany) was used for isolating total cellular RNA following the manufacturer’s instructions. Then, using a spectrophotometer, the RNA concentration was determined. The RNA was preserved at −80 °C. The cDNA was synthesized via a BioFACT 2step 2X RT-PCR Pre-Mix (Taq). The Applied Biosystems StepOnePlusTM Real-Time PCR System (Life Technologies, Carlsbad, CA, USA) was applied to evaluate all genes expressed in this study. For internal control, the 18s gene was evaluated to normalize the target mRNA expression. In Table 1, the primer sequences are shown. All of the experiments were conducted in triplicate, and the 2^−ΔΔCt^ method was utilized for quantifying the relative mRNA expression.

### 2.9. Cytokine Secretion Assay

Freshly obtained CD3^+^ T cells were grown in 24-well plates with mDCs and B7H7-silenced-mDCs in 1:5 ratios to investigate the potential of mDCs and B7H7-silenced-mDCs to enhance cytokine secretion in the presence of autologous T cells. Then 48 h after stimulation with DCs, the co-culture supernatants were harvested, and the amounts of IFN-γ, TGF-β, and IL-4, were quantified through commercial ELISA kits (R&D Systems, Minneapolis, MN, USA). IL-12 and IL-10 levels were also quantified in mDC and B7H7-silenced-mDC supernatants utilizing ELISA kits (R&D Systems, Minneapolis, MN, USA).

### 2.10. Statistical Analysis

The raw data were analyzed using GraphPad Prism v8.0.2 (GraphPad Software, San Diego, CA, USA). Data comparisons between two groups were performed using the Student’s *t*-test, while comparisons among several groups were conducted using one-way ANOVA tests. Each parameter was assessed in triplicate, and each independent sample’s mean of triplicates was used in statistical comparisons between groups. The results from each group were presented as mean ± SD. A significance cut-off of *p*-value ≤ 0.05 was used to determine statistical significance. (ns: not significant; *: *p* ≤ 0.05; **: *p* ≤ 0.01; ***: *p* ≤ 0.001; and ****: *p* ≤ 0.0001).

## 3. Results

### 3.1. Transfection of siRNA Resulted in Diminished B7H7 Molecule’s Gene Expression in DCs

**Figure 2:**
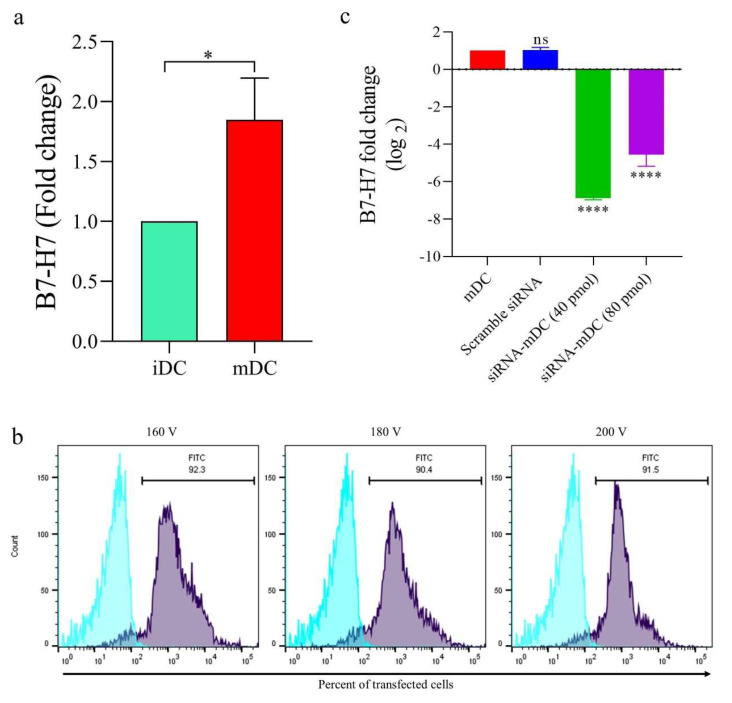
Transfection with siRNA significantly suppressed B7H7 gene expression in mDCs. (**a**) Compared to the iDCs, mDCs showed significantly higher levels of B7H7 mRNA expression. (**b**) All three different pulse voltages showed above 90% of FITC_labeled control siRNA transfection into the mDCs. The viability for transfected and untransfected mDCs was more than 90%, determined using the Trypan blue exclusion test. (**c**) Compared to the other concentrations, 40 pmol of B7H7 siRNA showed the most significant effect in suppressing B7H7 mRNA expression after 72 h of transfection in comparison with untransfected mDCs; (ns: not significant, *: *p* ≤ 0.05, and **** *p* ≤ 0.0001). Abbreviations: siRNA: small interfering RNA, iDCs: immature dendritic cells, mDCs: tumor cell lysate_loaded mature dendritic cells.

First, the B7H7 expression on DCs was confirmed using qRT-PCR analysis. Accordingly, in comparison to iDCs, the mRNA of B7H7 in mDCs was significantly upregulated (Figure 2a, *p* ≤ 0.05). Subsequently, mDCs were transfected with various pulse voltages to determine the optimal voltage for transfection (160, 180, and 200 V). The transfection rate was estimated to be approximately 90% in all DC groups, with no noticeable differences between them (Figure 2b); hence, to diminish stress in DCs during the transfection process, the lowest voltage (160 V) was used as the optimal pulse voltage. Moreover, after the transfection of the scramble siRNA and B7H7-siRNA into mDCs in various concentrations, to demonstrate the efficiency of siRNA-mediated B7H7 mRNA reduction, RNA was isolated from the DCs 72 h after electroporation, and B7H7 mRNA levels were assessed via qRT-PCR. At 72 h of incubation time, 40 pmol of B7H7 siRNA, compared to 80 pmol, decreased B7H7 mRNA expression significantly in transfected cells compared to untransfected mDCs (Figure 2c, *p* ≤ 0.0001). There was no significant difference between the scramble siRNA group and the untransfected mDCs. Based on these results, the following investigations were carried out utilizing a 40 pmol B7H7-siRNA as the optimal dosage and a 160 V optimal transfection voltage.

### 3.2. DCs’ Maturation and Activation Are Augmented by siRNA-Mediated B7H7 Knockdown

**Figure 3:**
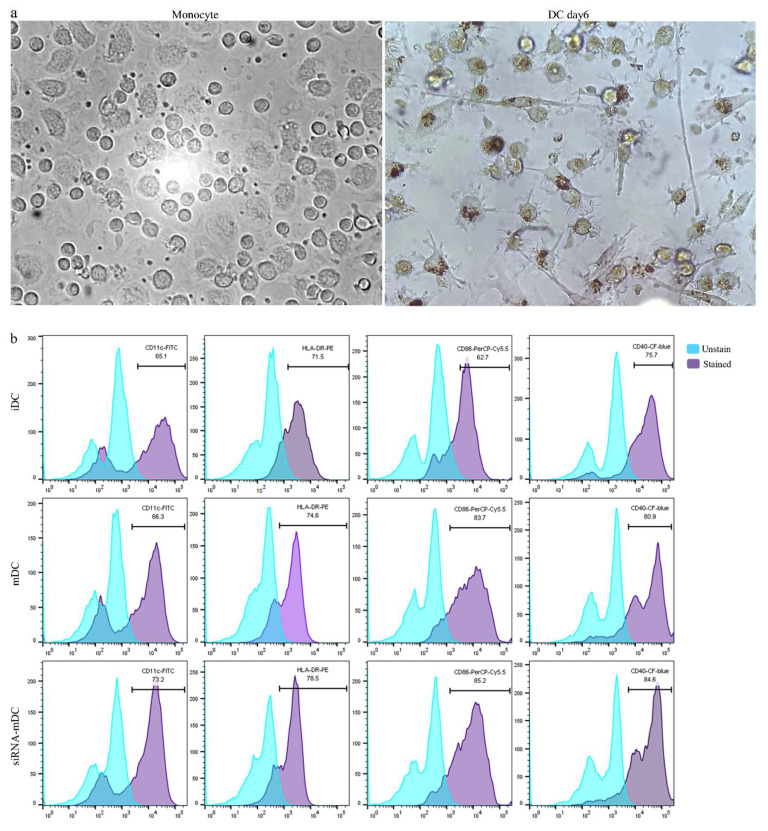

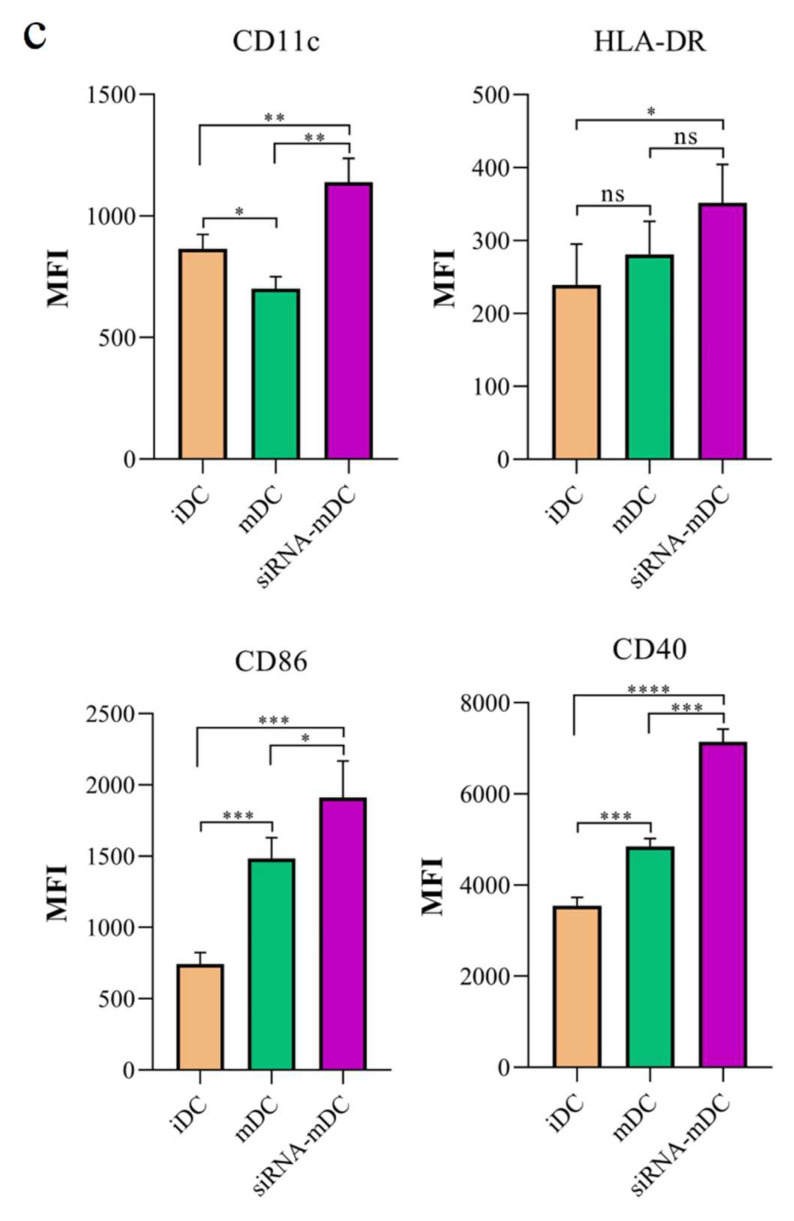

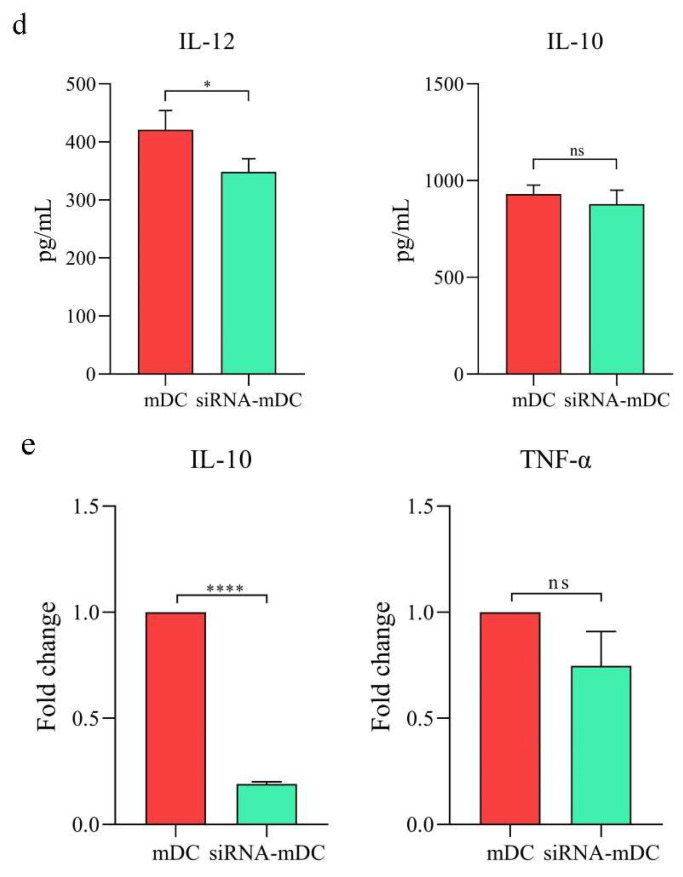
B7H7 silencing boosted DCs functional characteristics. (**a**) Morphological changes include the appearance of sharp dendrites during in vitro differentiation of monocytes into DCs. (**b**) Flow cytometry plots (representative of all samples) indicate the percentage of stained cells for the surface markers, including CD11c, HLA-DR, CD40, and CD86 in iDCs, mDCs, and B7H7-silenced mDCs. (**c**) Comparison between the amounts of MFI for CD11c, HLA-DR, CD40, and CD86 markers between iDCs, mDCs, and B7H7-silenced mDCs. (**d**) ELISA measured the concentration of IL-12 and IL-10 cytokines in the cell culture supernatants. (**e**) qRT-PCR was employed to evaluate the expression of TNF-α and IL-10 genes; (ns: not significant, *: *p* ≤ 0.05, **: *p* ≤ 0.01, ***: *p* ≤ 0.001, and ****: *p* ≤ 0.0001). Abbreviations: iDCs: immature dendritic cells, mDCs: Tumor cell lysate-loaded mature dendritic cells, MFI: Median fluorescence intensity, ELISA: Enzyme-linked immunosorbent assay, qRT-PCR: Quantitative reverse transcription polymerase chain reaction, TNF-α: Tumor necrosis factor-α.

The morphological differences found throughout in vitro monocyte culture, and in the generated DCs, were revealed using microscopic analysis (Figure 3a). DC immunophenotypes were then assessed via staining with antibodies that are specific to markers associated with DC maturation and antigen presentation. According to the flow cytometry results, CD11c, HLA-DR, CD86, and CD40 expressions were all detected in iDCs and mDCs, as was expected (Figure 3b). To investigate whether the electroporation of B7H7 siRNA into mDCs may influence their maturation and activation, the modifications in these markers’ surface expression between mDCs and B7H7-transfected mDCs were evaluated according to the median fluorescence intensity (MFI) as shown Figure 3c. B7H7 silencing in mDCs enhanced the surface expression of CD11c (*p* ≤ 0.01), CD86 (*p* ≤ 0.05), and CD40 (*p* ≤ 0.001) substantially in comparison to mDCs, but it had no significant impacts on the elevated levels of HLA-DR expression (Figure 3c). The B7H7 silencing impact on DCs’ function was further examined with cytokine secretion analysis. Consequently, the levels of IL-10 and IL-12 in the cell culture supernatants and the TNF-α and IL-10 mRNAs expression were assessed using ELISA and qRT-PCR, respectively. Despite the decreased production of IL-10 due to B7H7 inhibition, this decrease was not significant when compared to the mDCs (Figure 3d). Compared to mDCs, the knockdown of B7H7 resulted in a reduction in IL-12 levels in cell culture supernatants (Figure 3d, *p* ≤ 0.05). Moreover, based on the qRT-PCR findings, B7H7 inhibition leads to diminished IL-10 mRNA expression in mDCs (Figure 3e, *p* ≤ 0.0001), while this suppression had no significant impact on the TNF-α expression (Figure 3e).

### 3.3. B7H7 siRNA Transfected DCs Showed the Enhanced Capability to Stimulate T-Cell Responses

**Figure 4:**
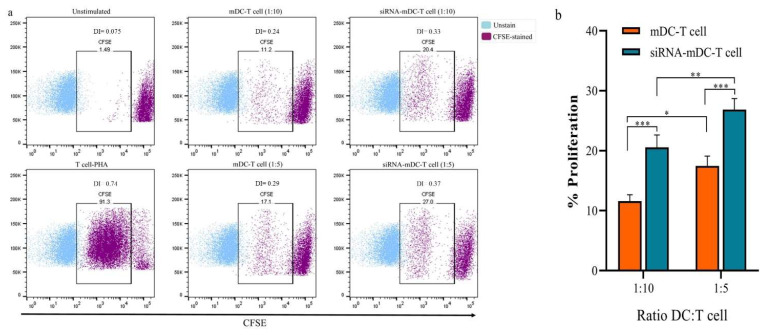
B7H7-silenced mDCs augmented the proliferation of autologous CD3^+^ T cells. (**a**) Flow cytometry plots (representative of all samples) indicating the percentage of CFSE-labeled autologous CD3^+^ T cells (via calculating the CFSE loss) stimulated with mDCs and B7H7-silenced mDCs at the ratios of 1:5 and 1:10 of DC: T cell. (**b**) Comparison of the percentage of proliferation between CD3^+^ T cells stimulated with mDCs and B7H7-silenced mDCs; (*: *p* ≤ 0.05, **: *p* ≤ 0.01, and ***: *p* ≤ 0.001). Abbreviations: mDCs: tumor cell lysate-loaded mature dendritic cells, siRNA-mDCs: B7H7-silenced mDCs, CFSE: Carboxyfluorescein succinimidyl ester, PHA: Phytohemagglutinin.

**Figure 5:**
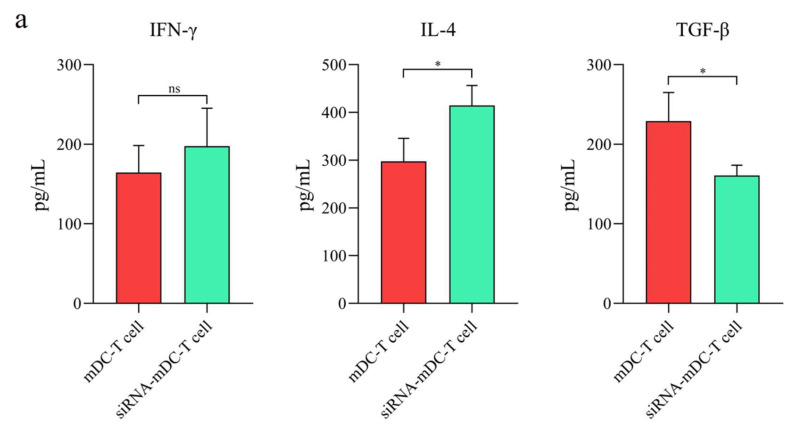

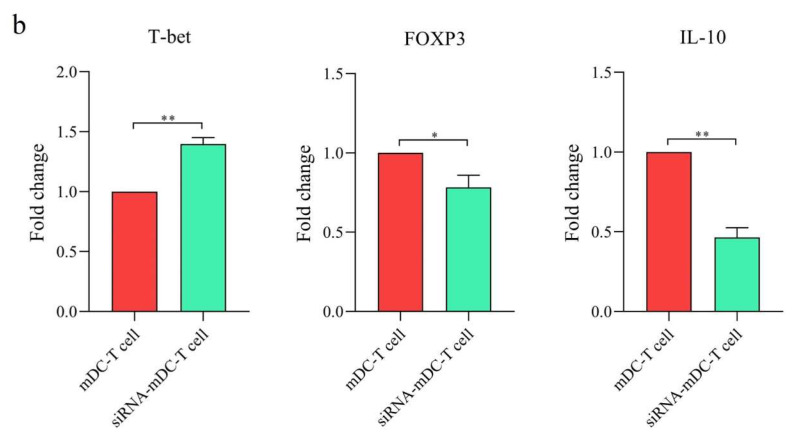
B7H7-silenced mDCs improved T cell-mediated effector functions. (**a**) IFN-γ, IL-4, and TGF-β cytokines’ production by autologous CD3^+^ T cells after co-culture with mDCs and B7H7-silenced mDCs evaluated by ELISA. (**b**) Comparison of T-bet, FOXP3 and IL-10 genes expression in CD3^+^ T cells following co-culture with mDCs and B7H7-silenced mDCs evaluated by qRT-PCR; (ns: not significant, *: *p* ≤ 0.05, and **: *p* ≤ 0.01). Abbreviations: mDCs: tumor cell lysate-loaded mature dendritic cells, siRNA-mDCs: B7H7-silenced mDCs, IFN: interferon, TGF: transforming growth factor, ELISA: enzyme-linked immunosorbent assay, T-bet: T-box protein expressed in T cells, FOXP3: Forkhead box P3, qRT-PCR: quantitative reverse transcription polymerase chain reaction.

In the subsequent assessments, CD3^+^ T lymphocytes’ cytokine production and expansion were analyzed to assess whether the transfection of B7H7 siRNA into mDCs would influence their capability to develop T cell-mediated responses. As mentioned in the methods section in detail, the co-culture test was carried out using mDCs and B7H7-inhibited mDCs as stimulators and CFSE-labeled autologous CD3^+^ T cells as responder cells in 1:5 and 1:10 ratios to determine the proliferation of CD3^+^ T cells. The findings showed that, in comparison to 1:10, the ratio of 1:5 led to higher CD3^+^ T lymphocyte expansion in all subgroups (Figure 4a,b, *p* ≤ 0.05). Additionally, B7H7-silenced mDCs had a superior potential to trigger CD3^+^ T cell proliferation than mDCs in both 1:10 and 1:5 ratios (Figure 4a,b, *p* ≤ 0.001). By analyzing the quantities of TGF-β, IL-4, and IFN-γ secreted from CD3^+^ T cells in the supernatant of T cells co-cultured with DCs, we could then examined the possibility that B7H7 silencing in mDCs would affect the cytokine production profiles of CD3^+^ T cells. Compared with the T cell/mDCs co-culture, the levels of IL-4 (Figure 5a, *p* ≤ 0.05) in the co-cultures of autologous T cells and B7H7-inhibited mDCs were significantly increased, while the quantities of TGF-β (Figure 5a, *p* ≤ 0.05) were significantly diminished. Compared to the T cell/mDCs co-culture, more IFN-γ was secreted from CD3^+^ T cells co-cultured with B7H7-suppressed mDCs, but there was no significant difference (Figure 5a). The results are consistent with increased T-bet mRNA expression in T cells isolated from T cell/B7H7-silenced mDCs co-culture compared to T cell/mDCs co-culture (Figure 5b, *p* ≤ 0.01). FOXP3 (Figure 5b, *p* ≤ 0.05) and IL-10 (Figure 5b, *p* ≤ 0.01) mRNA were revealed to be noticeably decreased in T cells co-cultured with B7H7-negative-mDCs compared with T cell/mDCs co-culture.

## 4. Discussion

Dendritic cells (DCs) are specialized cells that present tumor antigen/MHC complexes to other cells, such as natural killer (NK) cells, B cells, and naive and memory T cells [33], and anti-tumor immunity is regulated and coordinated by them. By capturing, processing, and presenting antigens to T cells, they trigger adaptive immune responses and participate in T cell-mediated anti-cancer immunity [33]. The therapeutic anti-cancer immune responses are increased both in vitro and in vivo through vaccination with tumor lysates-loaded DCs. These vaccines are effective anti-tumor treatments that can exploit the immune system’s strength and specificity for therapeutic purposes in treating various malignancies [34,35,36]. Based on recent investigations, several challenges must be overcome before DC-based immunotherapy can be effectively used to treat solid tumors. Limitations in developing an efficient DC vaccine include poor infiltration at the target site, unfavorable microenvironment conditions that render them ineffective, and diminished cytotoxic activity within solid tumors. To address these issues, innovative techniques for cell isolation, stimulation, and expansion need to be developed [37]. A patient’s diminished anti-cancer immunity due to their immunosuppressive TME is one of the most significant challenges [33]. 

In this regard, it remains challenging for DC-based cell therapy to be effective because of immunosuppressive factors in the tumor milieu, mainly inhibitory ICPs. A tumor cell’s ability to escape anti-cancer immunity can be attributed to the overexpression of inhibitory ICPs on DCs within the tumor setting [16]. Enhancing the effectiveness of DC-based immunotherapy by mitigating the functions of immunosuppressive molecules could be a promising approach [38]. 

As a recently identified B7 family ligand, B7H7 and its receptor CD28H can be expressed in human immune cells [23,24]. B7H7 is not expressed by iDCs or resting B and T cells in the human immune system but is strongly expressed by macrophages and monocytes [29]. Upon maturation of iDCs, the expression of B7H7 is augmented [23]. No studies have revealed the role of the B7H7 molecule expressed by DCs and its correlation to the function of T cells. Consequently, we aimed to determine whether the enhanced expression of B7H7 by DCs involved inhibiting T cells’ function, i.e., cytokine secretion and proliferation. We transfected siRNA to inhibit B7H7 expression in GC cell lysate-loaded DCs and assessed the impact of this suppression on the generation of robust anti-cancer immune response. 

According to our findings, B7H7 was expressed at considerably higher levels in mDCs than in iDCs (Figure 2a). Furthermore, to confirm the effectiveness of the siRNA-related downregulation of B7H7 mRNA, the expression of these molecules was analyzed using qRT-PCR. Following 72 h of incubation after electroporation with 40 pmol of B7H7 siRNA at 160 V, B7H7 mRNA expression in transfected mDCs was substantially downregulated compared to untransfected mDCs (Figure 2b). Following the knockdown of B7H7 in mDCs, we studied the stimulatory effect of this inhibition on mDCs’ maturation, activation, and function by evaluating surface molecule expression profiles and cytokine assay. As shown in Figure 3c, the surface expression of CD11c, CD86, and CD40 was significantly increased in mDCs with B7H7 knockdown compared with untransfected mDCs. However, the upregulation of HLA-DR in transfected mDCs was not considerably influenced by B7H7 inhibition (Figure 3c). Moreover, although IL-10 secretion was diminished in B7H7-silenced DCs, this diminishment was not significant (Figure 3d). In addition, in opposition to what we would have expected, B7H7 silencing decreased IL-12 concentration in cell culture supernatants compared to mDCs (Figure 3d). This conflicting result needs more investigation. A study held by Hai-Yu Xiong et al. revealed that IL-12 could be considered a B7H1 expression regulator in ovarian cancer-associated macrophages via affecting NF-κB signaling. While the relationship between IL-12 and B7-H7 has not been established, its association with other B7 family members may provide insights for further investigations [39].

The qRT-PCR results indicated that B7H7 knockdown downregulated the expression of IL-10 mRNA in mDCs but had no significant impact on TNF-α expression (Figure 3e). Given that antigen-loaded DCs can activate T lymphocytes, we investigated whether the ability of GC lysate-pulsed DCs to induce T cell immunity might be enhanced by the siRNA-mediated suppression of the B7H7 molecule. Based on the co-culture assay findings, CD3+ T cells co-cultured with B7H7-silenced GC cell lysate-pulsed DCs showed enhanced expansion compared to those primed with GC cell lysate-loaded DCs (Figure 4b). Additionally, the silencing of B7H7 in GC cell lysate-loaded DCs diminished TGF-β and enhanced IL-4 secretion from CD3^+^ T cells, while the increased IFN-γ levels were not significantly associated with the B7H7 inhibition; this may be the consequence of diminished IL-12 secretion from B7H7-silenced mDCs (Figure 5a). In addition, the levels of IL-10 mRNA in co-cultured CD3^+^ T cells with B7H7 silenced GC cell lysate-pulsed DCs were significantly downregulated (Figure 5b). The mRNA levels of T cells’ transcription factors, T-bet (T-box transcription factor TBX21; a T helper 1-associated marker), and FOXP3 (a regulatory T cell-associated marker) were then evaluated for further analysis. In addition to activating the genetic programs for the Th1 lineage, T-bet also inhibits the genetic programs for the Th2 and Th17 lines. Thus, T-bet defines the lineage-determining transcription factor of the Th1, which is responsible for differentiating it from naive Th precursors [40]. Additionally, by increasing chromatin accessibility at IFN-γ genes and reducing CpG methylation, T-bet promotes transcription of a group of genes crucial for Th1 cell activity, including IFN-γ and the chemokine receptor CXCR3 [41]. According to our results, compared to CD3^+^ T cells primed with GC cell lysate-loaded DCs, downregulated FOXP3 and upregulated T-bet mRNA levels were detected in CD3^+^ T cells co-cultured with B7H7-silenced-mDCs (Figure 5b).

**Figure 6:**
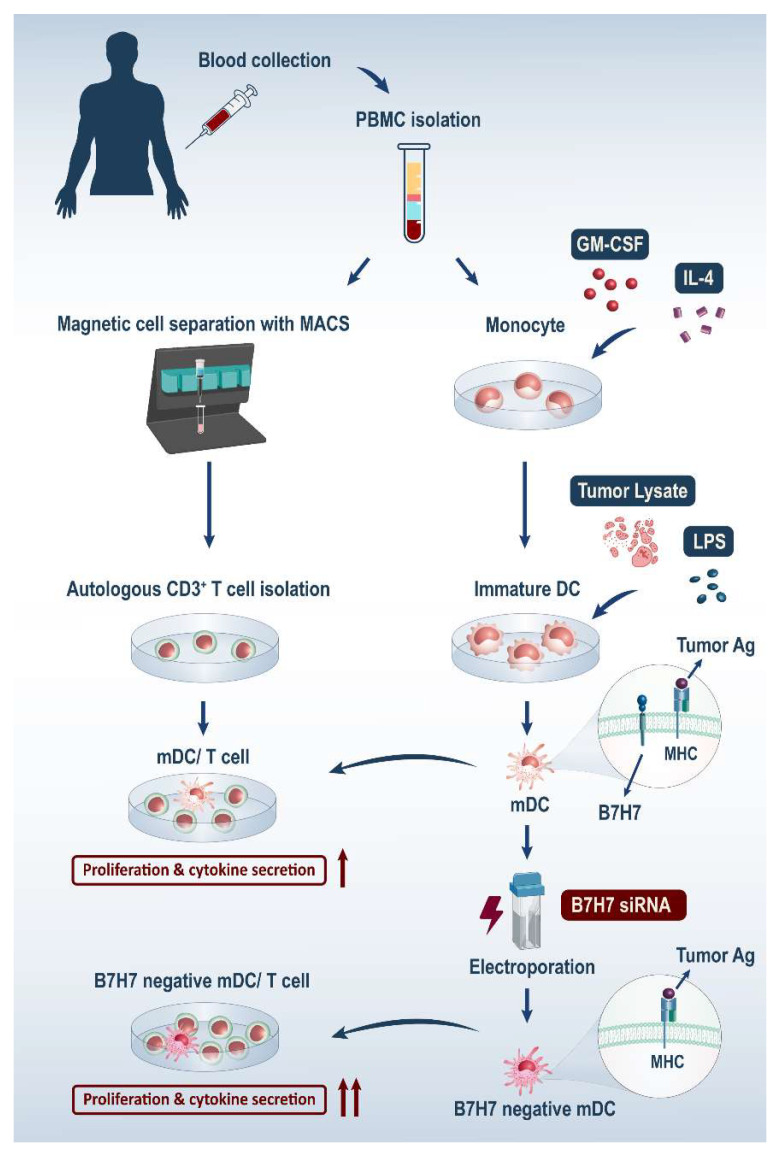
B7H7 molecules’ targeting in mDCs promotes autologous T-cell responses. siRNA-mediated B7H7 gene silencing in mDCs enhances their stimulatory capacity. As a result, co-culture of these B7H7-negative mDCs with autologous CD3^+^ T cells improves T-cell mediated responses. Abbreviations: mDC: tumor-lysate-pulsed mature dendritic cell, siRNA: small interfering RNA, PBMC: peripheral blood mononuclear cell, GM-CSF: granulocyte–macrophage colony-stimulating factor, LPS: Lipopolysaccharide, MHC: major histocompatibility complex, Ag: antigen, MACS: magnetic-activated cell sorting.

As previously stated, no investigation has shown the role of the B7H7 molecule expressed in DCs and its correlation with T-cell activity. However, several studies on DC-based cell therapy have been performed to improve DCs’ ability to trigger immune responses against malignancies via the inhibition of inhibitory ICPs expressed in DCs. In this regard, it has been demonstrated that blocking programmed death-ligand 1 (PD-L1) promotes DC maturation and proliferation in a humanized SCID model of breast cancer [18]. According to their results, PD-L1 silenced DCs augment T cell proliferation and IFN-γ secretion [18]. In another investigation, PD-L1 knockdown was related to the upregulation of CD86 and HLA-DR in DCs, which was similar to our findings [42]. Furthermore, PD-L1 negative DCs demonstrated significant potential to stimulate T cell expansion and IFN-γ secretion [42]. Similar to our results, the inhibition of PD-L1 in DCs decreased the TGF-β and IL-10 levels in DC/T cell co-culture supernatants [42]. Van der Waart and colleagues have shown that siRNA-based knockdown of PD-L1/2 in DC vaccines boosted CD8^+^ T-lymphocyte expansion [43]. It has been demonstrated that, in transplanted cancer patients, the PD-L1/2-silenced DCs pulsed with minor histocompatibility antigen (MiHA) mRNA exhibit an improved stimulatory capability and amplified ex vivo antigen-specific CD8^+^ T cell immunity [44]. According to Oh and colleagues, PD-L1 silencing in DCs can lead to stronger anti-tumor CD8^+^ T-cell response and a marked reduction in tumor growth [45]. Furthermore, it has been shown that PD-L gene knockdown, particularly the combination PD-L1 and PD-L2 suppression, led to increased CD4^+^ T cell expansion [46] (Figure 6).

The immunosuppressive action of the B7 family seems to be a common mechanism in many types of tumors; therefore, our finding can be utilized for other types of cancer. Previously, B7 family expression was studied in the prognosis of acute myeloid leukemia (AML), which revealed that the mRNA levels of B7 family members were altered in numerous AML patients. Higher expression of B7 members was correlated with poor prognosis in AML and worse overall survival [47].

While siRNA holds significant potential for effectively inhibiting gene expression, its broader application is hindered by challenges such as low stability, suboptimal pharmacokinetics, and potential off-target effects [48]. Additionally, even though electroporation is a successful way of delivering siRNAs to human cells resistant to lipid-based transfection techniques [49], this approach has disadvantages. It can reduce cell viability due to membrane disruption, trigger cellular stress responses, and limit scalability for high-throughput applications. It facilitates the transportation of molecules through nano-size pores in the membrane [50]. Therefore, there is a need for the replacement of other optimized siRNA transfection methods.

Future investigations should consider enhancing the efficacy of this approach by combining the lysate-activated DCs with other strategies. In line with this, Lianjing Zhao and colleagues investigated DCs loaded with the lysate of Newcastle Disease Virus-infected tumor cells to induce more potent anti-tumor immunity by augmenting the production of IL-2 and IFN-γ by T cells [51]. Similar to our study, Kohnepoushi et al. investigated GC lysates-activated DCs; however, they compared the efficacy of the tumor lysate in the soluble and encapsulated forms in nanoparticles. Their findings revealed that nanoparticle-encapsulated antigens could alternate anti-tumor T-cell response and shift them toward a Th1 pathway, potentially increasing DC vaccine potency in clinical settings. The experiment showed increased CD80, CD83, CD86, HLA-DR, IL-12, IL-10, and IFN-γ:IL-4 ratios. So, even antigen delivery methods for the same type of tumor cell lysates can influence the response [12].

On the other hand, the DC vaccine against malignancy may be a piece in a puzzle that needs other pieces to overcome the challenges. In this regard, using chimeric antigen receptor (CAR) activated T cells can have a complementary role in these challenges. For instance, Liu et al. showed a synergistic killing effect when combining PD-L1-CAR T cells with DCs loaded with lysates from colorectal cancer stem cells [52]. Assessing the in vivo effects of various immunotherapeutics, including immune checkpoints like B7H7, on T cell expansion and cytokine secretion in clinical contexts to determine treatment efficacy has been challenging. There is a pressing need to find solutions to these issues.

In a recent study by Bresser et al., a novel method named “Division Recorder” was introduced. This genetic-tracing approach reveals the historical proliferation extent of cell pools in vivo. Additionally, when combined with single-cell transcriptomics, they found that replicative history differentiates specific cell groups within the central memory T cells’ compartment [53].

## 5. Conclusions

We have identified a novel immunosuppressive role for the B7H7 molecule in DCs’ function for the first time. The results of our study suggest that B7H7 knockdown significantly enhances the maturation and stimulatory activity of DCs pulsed with GC cell lysate. The B7H7-silenced DCs can substantially boost co-cultured T-cells’ cytokine production and expansion. Taken together, B7H7 suppression in DCs may prove to be a practical approach for enhancing the capacity of DCs to trigger T lymphocyte responses and boost the potency of DC-based cell therapy in gastric cancer. In light of these results, this treatment approach is proposed to be examined further in preclinical studies.

## Figures and Tables

**Table 1:** List of primer and siRNA sequences.

Gene		Sequence
B7H7 siRNA	Sense Antisense	GCCAAGAAACAGCTTCCCATA
		TATGGGAAGCTGTTTCTTGGC
*B7H7*	F R	TCAGTCCTTGGATAGTGAGGTTC
		TCAGTCCTTGGATAGTGAGGTTC
*TNF-α*	F R	TTCTCCTTCCTGATCGTGGCA
		TAGAGAGAGGTCCCTGGGGAA
*IL-10*	F R	AGGAAGAGAAACCAGGGAGC
		GAATCCCTCCGAGACACTGG
*T-bet*	F R	TCTCCTCTCCTACCCAACCAG
		CATGCTGACTGCTCGAAACTCA
*FOXP*	F R	CAGCCAGTCTATGCAAACC
		GTCTTGTGTCAGTTTGAGGGTC
*18S*	F R	CTACGTCCCTGCCCTTTGTACA
		ACACTTCACCGGACCATTCAA

## Data Availability

The data presented in this study are available on request from the corresponding author.
